# Use of Finite Difference Time Domain Simulations and Debye Theory for Modelling the Terahertz Reflection Response of Normal and Tumour Breast Tissue

**DOI:** 10.1371/journal.pone.0099291

**Published:** 2014-07-10

**Authors:** Anthony J. Fitzgerald, Emma Pickwell-MacPherson, Vincent P. Wallace

**Affiliations:** 1 School of Physics, University of Western Australia, WA, Australia; 2 Electronic Engineering Department, Chinese University of Hong Kong, Hong Kong; Tufts University, United States of America

## Abstract

The aim of this work was to evaluate the capabilities of Debye theory combined with Finite Difference Time Domain (FDTD) methods to simulate the terahertz (THz) response of breast tissues. Being able to accurately model breast tissues in the THz regime would facilitate the understanding of image contrast parameters used in THz imaging of breast cancer. As a test case, the model was first validated using liquid water and simulated reflection pulses were compared to experimental measured pulses with very good agreement (p = 1.00). The responses of normal and cancerous breast tissues were simulated with Debye properties and the correlation with measured data was still high for tumour (p = 0.98) and less so for normal breast (p = 0.82). Sections of the time domain pulses showed clear differences that were also evident in the comparison of pulse parameter values. These deviations may arise from the presence of adipose and other inhomogeneities in the breast tissue that are not accounted for when using the Debye model. In conclusion, the study demonstrates the power of the model for simulating THz reflection imaging; however, for biological tissues extra Debye terms or a more detailed theory may be required to link THz image contrast to physiological composition and structural changes of breast tissue associated with differences between normal and tumour tissues.

## Introduction

### Background

A number of papers have been published on the applications of terahertz (THz) technology for the detection and diagnosis of cancer [1]–[3] including skin cancer [4]–[6] cervical cancer [7], colon cancer [8], [9] and breast cancer [10]–[12]. This focus on tissue imaging using THz has progressed the technology from the laboratory bench-top to the clinic through, for instance, the development of a prototype endoscope [13] and a handheld THz imaging probe [14] for intra-operative use during breast cancer surgery to assist in identifying regions of disease and ensure its complete removal.

To advance the detection of tumours in tissue using THz technology, data reduction and classification methods have been used based on characteristics of the pulse profile or shape [15], [16]. The inputs into the classification algorithms are based on a set of parameters that are either heuristic in nature, i.e. selected due to observed differences between pulses reflected from normal and diseased tissues, or through principal component analysis (PCA), i.e. statistically significant differences between the pulse profiles from different tissue pathologies. The usefulness of the pulse profile characteristics in identifying tissue pathology is highlighted by the classification accuracy of 92% using PCA for breast cancer [15] and around 90% with colon cancer [16]. The reason for this high discrimination ability is often attributed to differences in the tissue water content which provides contrast between normal tissue and tumours, however this has not been fully explored. In addition, it has been suggested by others for example, Png *et al* [17] and Sy *et al* [18] that water is not the only source of contrast and other tissue pathology features may contribute, although it is not clear how at this stage.

Here, we review the application of Double Debye theory in THz biomedical imaging and we build on previous simulation studies [19], [20] and attempt to develop a more comprehensive model for the interaction of THz radiation with breast tissue. We evaluate the use of double Debye theory for modelling breast tissue by direct comparison of measured pulses and parameters from simulations and real breast tissue samples. The ability to model the changes would allow us to identify what contrast mechanisms contribute to THz images and classification of cancer, and most importantly, to link the pulse profile parameters to changes in the composition and physiology of breast tissue.

### Theory – double Debye and FDTD model

We have previously simulated the interaction of THz radiation (0.1–2 THz) with bulk water and skin tissues by using double Debye theory combined with finite difference time domain (FDTD) methods [21], [22]. An advantage of employing FDTD methods is that the model can be used to simulate both transmission and reflection geometries. As biological tissues have high water content, clinical measurements are only possible in reflection geometry [23], [24] thus Pickwell used a double Debye approach, and transmission spectroscopy data, to successfully simulate the THz reflection pulses of human skin that compared well with measured reflection pulses.

Here we use a similar but updated fitting approach and apply it to breast tissue. The inputs into the FDTD model are double Debye parameters calculated from spectroscopy data. The Debye theory is an accepted method of characterizing the temporal relaxation of frequency dependent local dielectric polarisation changes in a medium by coupling it to the local electric field strength [25]. In the THz regime, studies have shown that for liquid water there are two main time constants in action [26]–[28]. The faster one of these occurs as a result of single water molecules re-orienting and moving in the field. The slower time constant is a function of a collective structural mode, whereby tetrahedral “cages” of water molecules reorient under the changing field. By treating the two polarization decay rates independently, we use double Debye theory to model the slow (τ_1_) and fast (τ_2_) relaxation processes of water using the following equation:
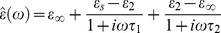
(1)Where ε*_S_* is the static dielectric constant, ε_∞_, is the limiting value at high frequency, and ε_2_ is an intermediate frequency limit.

As explained in Pickwell *et al* [21] the complex dielectric coefficient 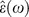 is related to the complex refractive index 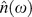 (Eqn. 2) explicitly, so that we may calculate the real and imaginary terms of 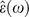 directly from the absorption coefficient and refractive index.
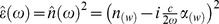
(2)

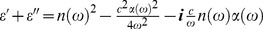
(3)


Through these equations, THz time domain spectroscopy data (absorption coefficient and refractive index) from excised tissue samples can be used to obtain the five characterizing double Debye values: ε_∞_, ε_S_, ε_2,_ τ_1_ and τ_2_. In the paper by Pickwell *et al* [19] Eqn. (2) was parameterised and by minimizing the difference between the real and imaginary parts of the model and the measured data (using a least squares approach) they found the best fit for the double Debye parameters. Here, we use a different formulation (Eqn. 3) which allows for the least square fitting of the double Debye parameters directly. In addition, the method used here focuses on improving the fit at lower frequencies as the work by Ashworth *et al* [29] show that the differences between breast tissue pathologies is greater at lower frequencies, with the maximum difference at 0.32 THz.

In this study, we use a similar approach, with the assumption that double Debye theory can be applied to breast tissues. Firstly, the transmission spectroscopy data from breast tissue is used to obtain the double Debye values, by fitting Equation 3. As this is different from what has been previously published we validate the method using liquid water. The FDTD model is used to simulate the reflected waveforms and these are compared with real data. The model is then used to generate simulated THz pulses for breast tissue with varying quantities of pathology (tumour, fibrous and adipose tissues) to compare with actual measured THz pulses from freshly excised breast tissues.

## Materials and Methods

### THz transmission spectroscopy

All spectroscopy measurements were performed in transmission using the TPI™spectra1000 (TeraView Limited, Cambridge, UK) previously described by Taday and Newnham [30]. The instrument generates pulses of broadband THz radiation in the range 0.05 THz to 4 THz with a spectral resolution of 0.03 THz in rapid scanning mode (30 scans in 1 second).

The signals measured by the THz spectroscopy system are time domain waveforms that are directly proportional to the electric field. Frequency spectra are obtained in the THz range by Fourier transformation of the time domain waveforms. As it is possible to recover both phase and amplitude information the frequency dependent refractive index of the sample, *n* and absorption coefficient, *α* can be calculated. The two windows have flat parallel sides, and THz radiation is incident normally as a plane wave. Liquid water is highly absorbing and given the sample thickness multiple reflections are minimal and Fabry-Perot effects are neglected. Thus the electric field is related to *α* and *n* by
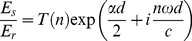
(4)Where *E_r_* and *E_s_* are the THz electric field of a reference and sample respectively, *d* is the sample thickness, *ω* the angular frequency of the radiation and *c* is the speed of light in a vacuum. *T(n)* is the Fresnel reflection loss at the surface.

Prior to measurement of each sample, a reference was recorded using the quartz windows with no spacer between them. To measure water, the liquid was placed into the sample holder with a 100 micron spacer which was then placed in the measurement compartment of the TPIspectra1000. THz radiation transmitted through the water was recorded in a rapid scanning mode. Thirty spectra were averaged and measurements were made at a room temperature of 22°C.

As described by Ashworth *et al* [29] to determine the absorption coefficient and refractive index of breast tissue, the tissue was placed in the sample holder with a spacer thickness chosen so that the windows could be gently pressed together to hold the tissue in place without deformation. The breast samples used were from patients undergoing breast surgery at the Breast Cancer Unit, Addenbrooke’s Hospital, Cambridge. Histopathologists at the same hospital analysed the sections of tissue to confirm regions of tissues that were normal, adipose and had tumour. Approval for the study was granted by the local Cambridge Research Ethics Committee, Addenbrooke’s Hospital, Cambridge UK. Signed informed consent, agreeing to research on tissue removed at the time of surgery, was obtained from all patients.

The spectroscopic values for pure normal, tumour and adipose (fatty) tissue were obtained by applying a volume correction for the percentage components of normal, tumour and adipose tissue in each sample. This volume correction was necessary since each sample included components of adipose, normal or tumour in different ratios. The volume correction is more fully detailed in the Ashworth *et al* article [29].

**Figure 1:**
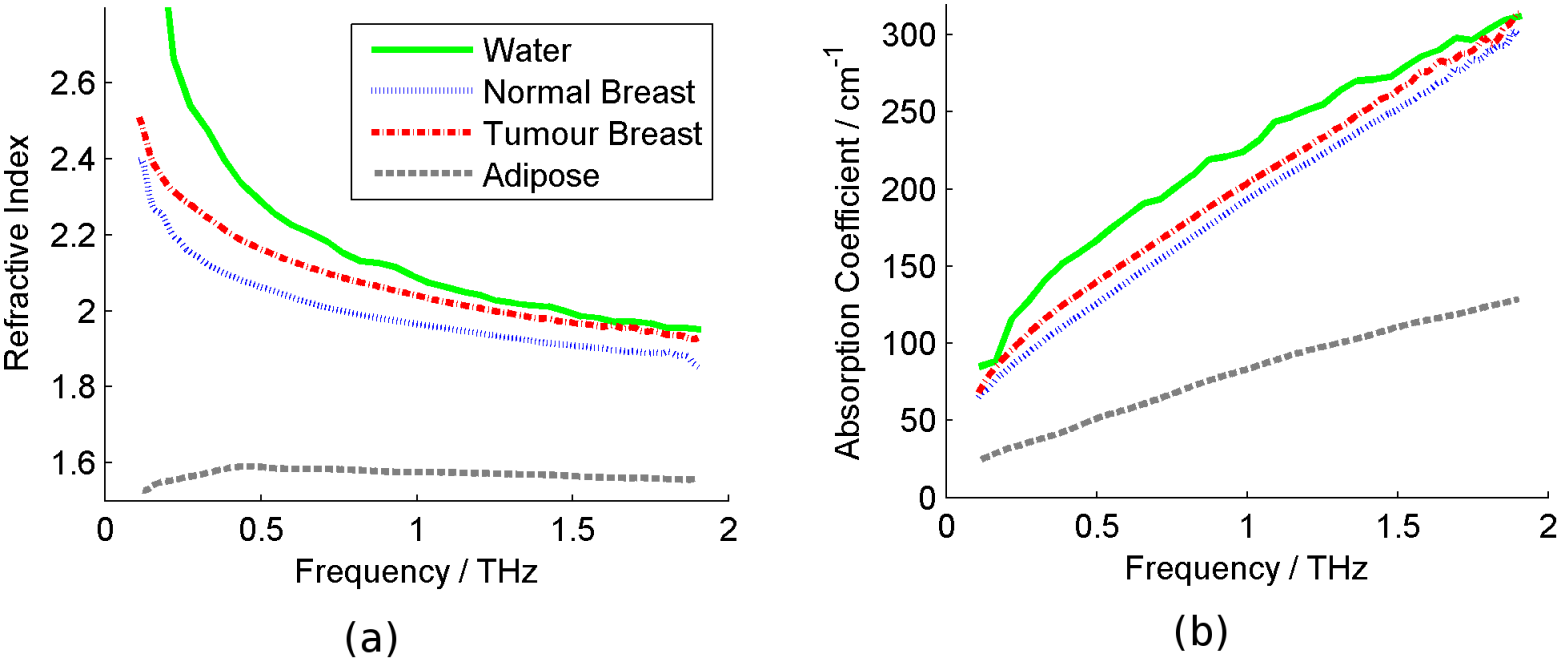
The terahertz spectroscopic properties for water and breast tissues. (a) Refractive index and (b) absorption coefficient for water (blue full line), normal breast tissue (green dotted line), breast tissue containing tumour (red dot-dash line) and adipose tissue (grey dashed line).

The frequency dependent absorption coefficient and refractive index values for water, pure normal and tumour breast tissue (Figure 1) determined from Equation 1 were used to calculate the double Debye values using the method described earlier. The mean double Debye values of all the samples were used in the FDTD simulation to model the THz reflection response of water, tumour and normal breast tissue.

### THz reflection measurements

Acquisition of THz reflection data was performed using the TPIimaga1000 (TeraView Ltd, Cambridge, UK). A full description of the system operation is given elsewhere [31]. The system uses photoconductive methods to generate and detect THz pulses reflected from the sample [24].

The broadband THz pulses are focused onto the top of a 2 mm thick z-cut quartz window where they are reflected at the interface of either quartz-air (reference pulse), or quartz-sample (sample pulse). An entire THz time domain waveform is acquired at each x-y point.

For the water reflection measurements, a well with walls of about 5 mm high was created on the quartz imaging window and was filled with water. Due to the high attenuation of THz radiation by liquid water, this is effectively a semi-infinite layer of water, so multiple reflections are negligible. To create a set of measurements over a defined area, the entire THz optics is raster-scanned in the x-y plane to collect a grid of data points which allowed for averaging and improvement of the signal-to-noise ratio. Again, all measurements were made at room temperature (22°C).

A THz impulse function was obtained from each raw THz waveform by deconvolving the system response and applying a double Gaussian filter to remove out of band noise as described in [32]. Each impulse function also referred to as a THz pulse, contained 512 time domain points which covered a time range of 33.8 ps. The same filter is used for the input to the FDTD simulation as discussed previously to match the bandwidth of experimental data.

**Figure 2:**
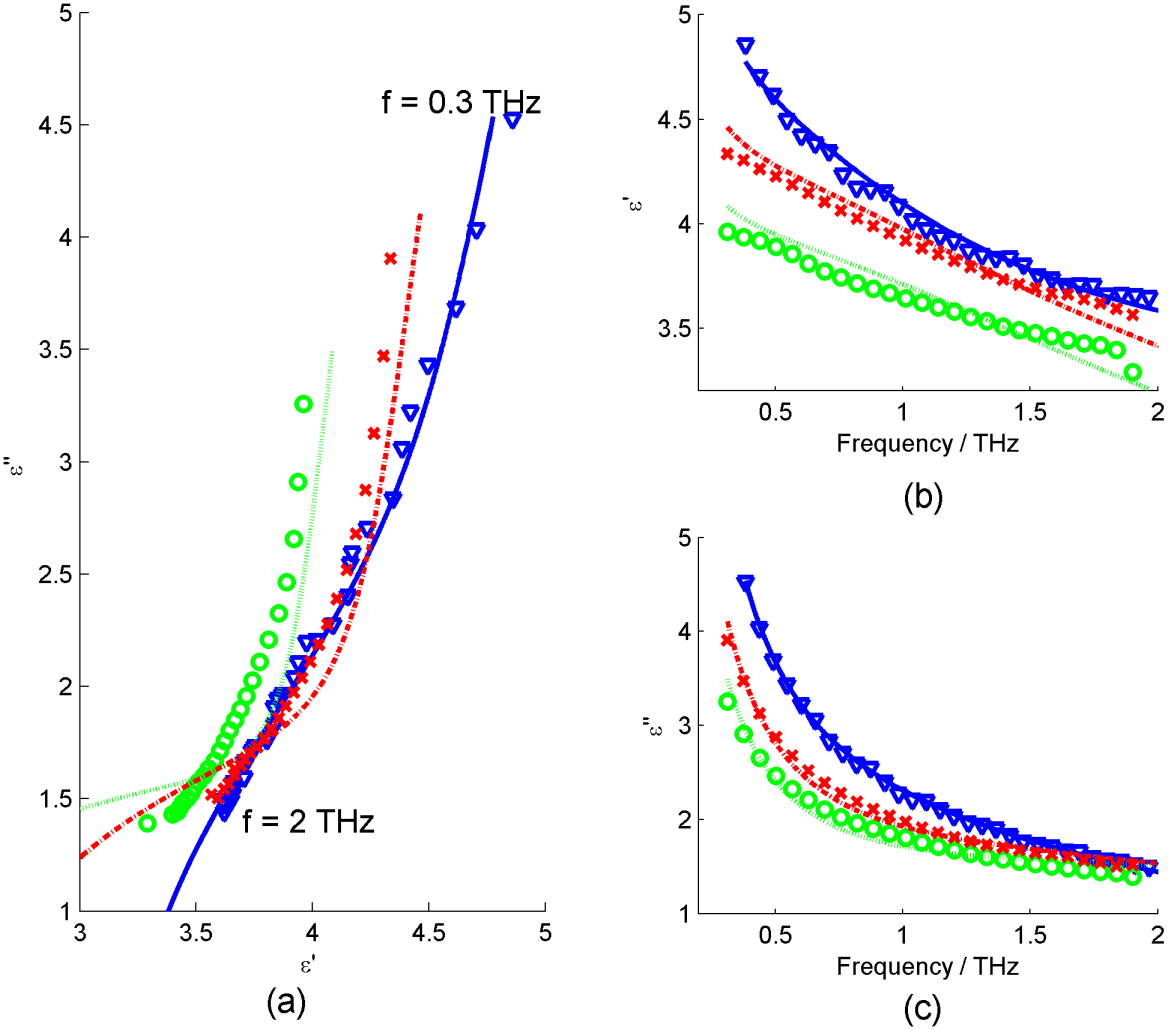
Simulation of liquid water using FDTD (full blue line) compared to measured water pulse (blue circles).

The mean reflected THz pulse from the water is compared in Figure 2 to the FDTD simulation of water using the double Debye values from the spectroscopy. For comparison, the two pulses were interpolated onto the same time domain grid and the pulse minima aligned in time. Goodness of fit of the simulated pulse to the measured pulse was determined using a χ^2^ test.

THz reflection measurements were made on breast tissue samples from 51 random, non-consecutive patients undergoing either wide local excision or mastectomy at Addenbrooke’s Hospital in Cambridge and Guy’s Hospital in London. Again, approval for the study was granted by the respective Local Research Ethics Committees. Signed informed consent, agreeing to research on tissue removed at the time of surgery, was obtained from all patients.The full details of this study are reported by Fitzgerald et al. [10]. The TPI Imaga1000 was again used to measure the THz reflection response of the samples.

**Figure 3:**
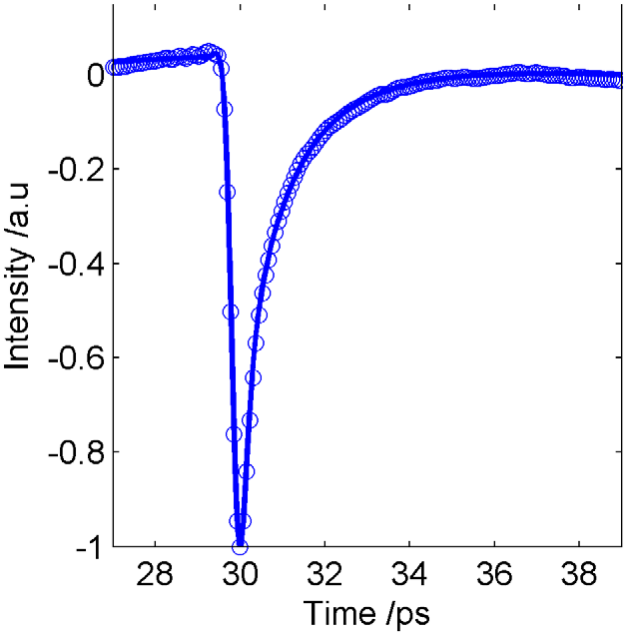
Comparison of the simulated and measured THz data. (a) Comparison of pulses for normal breast (simulated  =  green dotted line, measured  =  green dots), and breast tumour (simulated  =  red dot-dash line, measured  =  red crosses). (b) The differences between the measured and simulated pulses for normal breast (green dotted line) and tumour (red dot-dash line).

Waveforms from the normal tissues were pooled together for all samples and all patients and the mean impulse calculated from the full set of normal impulse functions. Similarly, for tumour, the mean impulse function was calculated from the collection of all patient and sample waveforms measured on tumour tissue. These mean impulse functions are compared with the simulated normal and tumour impulse functions from the FDTD simulation. The comparison is displayed in Figure 3.

### Double Debye model for water and breast tissue

In this paper we use a least squares regression approach implemented in Matlab (The Mathworks Inc, Natick, Massachusetts, USA) with the function lsqnonlin.m to minimise the sum of squares to fit the THz spectroscopy data by Equation 3. The double Debye values obtained can then be used for simulating THz responses by implementing them in the FDTD model.

**Figure 4:**
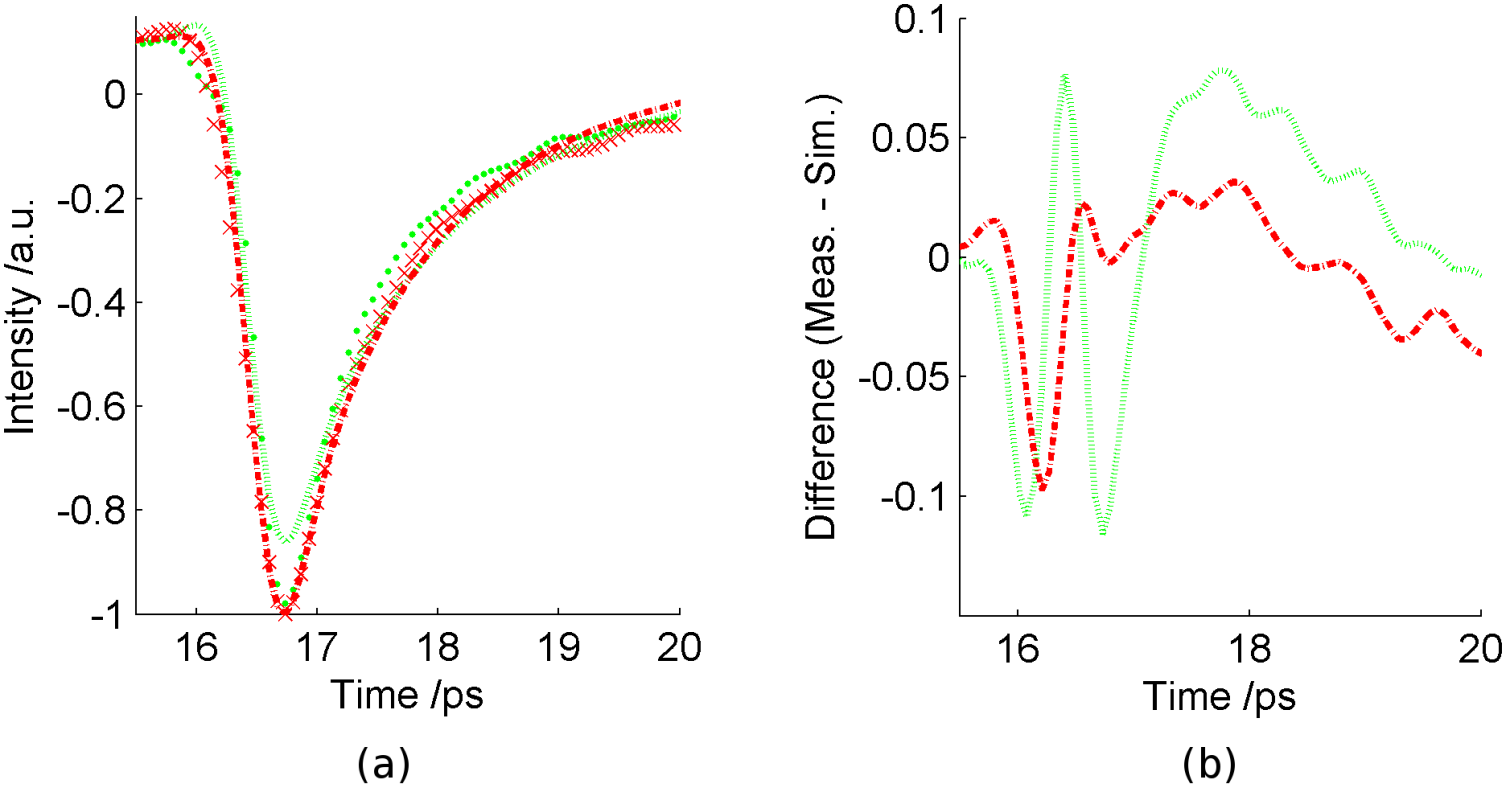
Plots of real (ε’) and imaginary (ε’’) terms of permittivity values calculated from spectroscopy data for water (blue triangles), normal breast (green circles) and tumour breast tissue (red crosses). (a) Shows the real versus imaginary permittivity, together with the best fit double Debye model determined from the least squares fitting method for water (full blue line), normal breast (green dotted line) and breast tumour (red dot-dash line), (b) Real terms of permittivity versus frequency and (c) Imaginary terms of permittivity versus frequency.

All double Debye values obtained by this method for liquid water and breast tissue are given in Table 1 of the results section. Figure 4 shows the fits to the real and imaginary terms of the complex permittivity for water and breast tissue.

**Table 1:** Double Debye values determined from the least squares fitting method.

	ε_S_	ε_2_	ε_∞_	τ_1_	τ_2_
Water [26]	78.4	4.9	3.5	8.2	0.18
Water [21]	78.8	6.6	4.1	10.6	0.18
Water - This work	78.4	4.8	3.2	8.0	0.18
Breast - normal	76.5	3.9	2.1	10.3	0.07
Breast - tumour	77.9	4.3	2.5	9.1	0.08

The values calculated in this paper for water are much closer to those values found by Kindt *et al* [26] than those of Pickwell *et al* [21].

### FDTD Simulations using double Debye values

The use of FDTD modelling using double Debye theory to simulate liquid water and biological tissue has been explained in a number of papers [21], [22], [33]. We use this same FDTD model, with input Double Debye values derived to simulate the propagation and reflection of THz electromagnetic radiation from liquid water and breast tissue. The FDTD model was programmed to directly replicate the THz reflection measurements of these modelled samples. For this study, we used a cell step size in the model of 1 µm, to maintain the frequency representation.

The electric field input into the FDTD simulation was chosen to correspond to the double Gaussian filter used in the image acquisition (imaging of liquid water and breast tissue). Once the double Debye parameters for the system to be modelled were calculated they were entered into the simulation along with the values defining the system to be modelled. By interpolating between cells in the lattice, effectively a continuous refractive index profile is formulated for a given system. With this input information the simulation then generates a reflected THz waveform that can be directly compared to measured waveforms from the same geometry.

Impulse functions from the FDTD model are compared to those reflection measurements of liquid water (Figure 2) and breast tissue (Figure 3).

### Comparison of pulse parameters for simulations and measurements for water and breast

**Figure 5:**
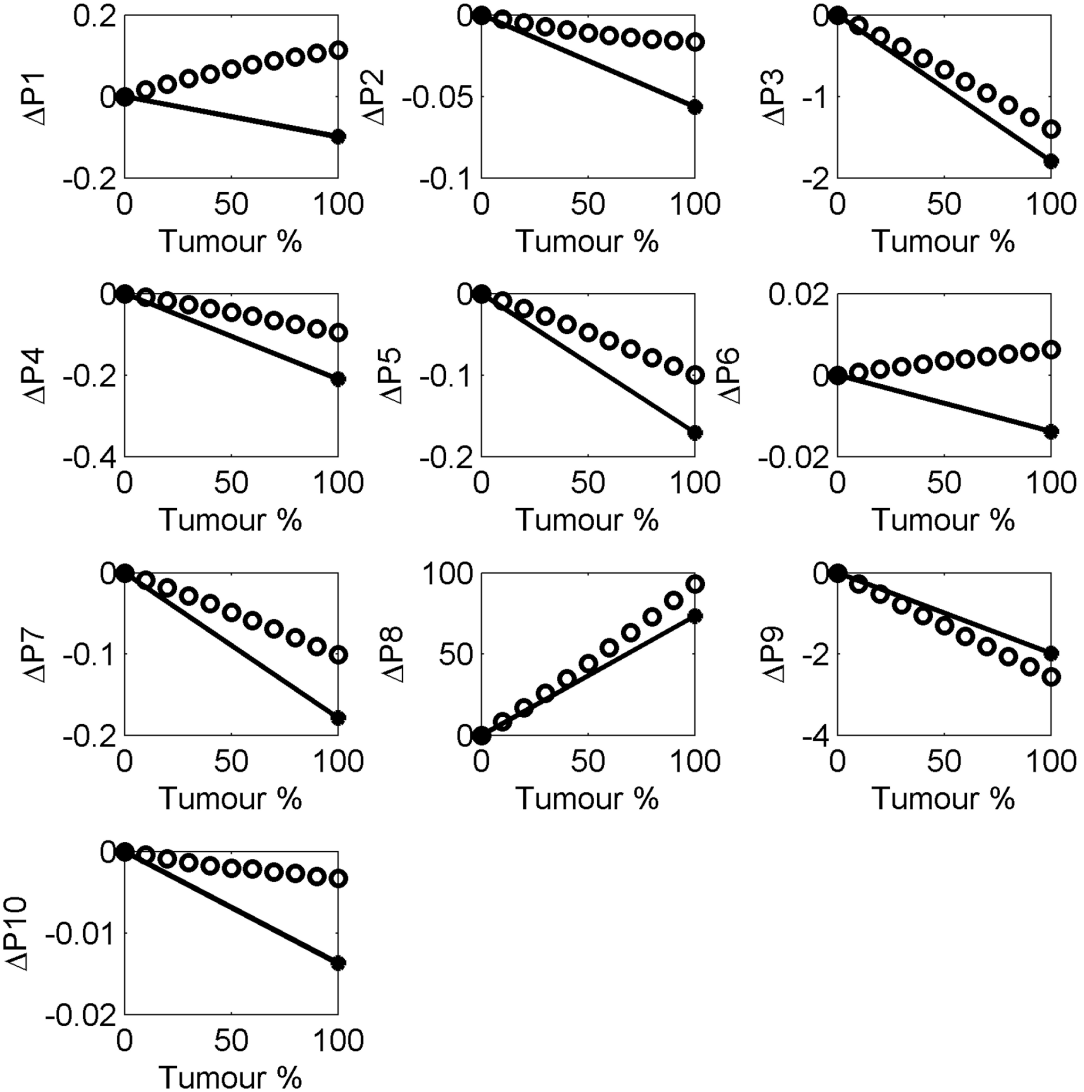
Change in the THz pulse parameter values as a function of the percentage of tumour. The values for normal breast were used as the baseline and the changes in the parameters were calculated as a function of the proportion of tumour, showing measured breast tissue (black dots, black line) and FDTD simulated breast response (black open circles).

Previous work has used heuristic parameters [15] to classify pulses from THz images of breast tissue as normal or tumour. In this study we investigate the same 10 parameters (see Table 2) and observe how the parameter values change with simulated cases of normal and tumour using the FDTD simulations. These parameter values are compared to the parameter values for the actual measured reflection pulses from the normal and tumour breast tissue from the breast study. For comparison purposes, we have investigated how the parameter values change in the simulation as the double Debye values go from normal values to tumour values, in steps of 10%. On this basis, normal is represented as 0% and tumour is 100%. The pulses were then simulated for each of the 11 cases, from normal (with 0% tumour properties), through 9 cases of increasing 10% of the double Debye values of normal to tumour, up to tumour, with 100% of the tumour Debye values. Parameters were then derived from each of these simulated pulses. These parameter values are compared in Figure 5 to the parameter values obtained from the measured mean normal and tumour breast pulses.

**Table 2:** The 10 time domain and frequency domain THz parameters from Fitzgerald et al [15].

Parameter	Expression	Feature Description
P1	FWHM	Full width half maximum of theabsolute magnitude of the pulse, |E_min_|.
P2	W@t (0.3E min)to T_Emin_	Time width of the pulse from the timeat which the pulse amplitude is initiallyat the fraction 0.3 of E_min_ to the timeT_Emin_.
P3	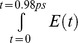	The integral area of amplitude of thepulse from T_Emin_ to t = 0.98ps
P 4	E = a_1_t+a_2_	The intercept, a_2_, of the linearregression fit from t = −0.26 ps to t = −0.66 ps.
P 5	E = a_1_t^2^+a_2_t+a_3_	Coefficient a_1_ from quadratic fit of theminimum section of the pulse from t = −0.20 ps to t = 0.20 ps.
P 6	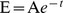	The coefficient λ from the exponentialcurve fit to the section from t = 0.66 psto t = 1.31 ps.
P 7	A(t = –0.26ps)	Amplitude of the pulse at time index t = −0.26 ps.
P 8	PS(f = 0.15 THz)	Power in spectrum at frequency = 0.15THz.
P 9	Re(FFT(f = 0.15 THz)	Real part of FFT as at frequency = 0.15THz.
P 10	Y = a_1_f+a_2_	Gradient, a_1_ of linear fit to logarithm ofthe power spectrum f = 0.15 THz tof = 1.50 THz.

Note that times given in picoseconds relate to the time T_Emin_, the time at which the main pulse is a minimum, E_min_.

## Results

### Double Debye Model

The double Debye values for liquid water and for breast tissue are given in Table 1. Liquid water has been well studied in the THz regime and the values from this work are compared with existing values in the literature. The Debye values for water calculated from this work agree very well with those in the literature [26]. Values of ε_S_ and τ_2_ agree precisely, while the least accurate term ε_∞_ deviates by about 8.5%. The accuracy of these Debye values indicates that the input into the FDTD simulation is accurate, which is positive from the modelling point of view in producing a pulse that closely matches the imaged water pulse (see Figure 2).

For breast tissue, it is clear from the Debye values that there is a difference in the THz response of normal and tumour tissue, with ε_∞_ differing by nearly 20%. The other terms vary by between 2% for ε_S_ and 15% for τ_2_. These differences indicate that the FDTD simulation pulses should show differences in amplitude and shape, since the double Debye values range so markedly. The simulated pulses are compared in Figure 3 together with the averaged pulses.

Figure 2 shows a very close agreement between the water pulse from the THz imaging and the impulse function from the FDTD simulation. The χ^2^ test resulted in a probability of p = 1.00, showing just how similar the pulses are.

As expected from the variation in the double Debye values, the simulated normal and tumour responses differ from each other (Figure 3). When compared with the simulated tumour pulse, the simulated normal pulse has a smaller amplitude, and begins to decrease at a later time, is narrower overall and returns to the baseline more slowly. These aspects are apparent in the parameters, as shown in Figure 5.

Also shown in Figure 3 are the mean THz pulses from the breast reflection measurements. It appears that the tumour pulses for the reflection measurements and simulation agree quite well, in terms of shape and width, and this is supported by the χ^2^ probability of p = 0.98. A small difference is seen as the pulses return to baseline after 17.5 ps, where the deviation is larger, as shown by the difference graph in Figure 3b. For the normal pulses, the simulation deviates a little more from the measured. Figure 3b shows that the normal simulated pulse is different in shape especially around the main pulse and also in the return to baseline after the minimum. This is reflected in the χ^2^ goodness of fit probability p = 0.82 determined for the simulated and measured normal pulses.

Figure 5 shows the results for the change in pulse parameter values as the double Debye values in the FDTD simulations change from those of normal breast tissue, to that of tumour. We take 0% tumour, i.e. normal tissue, as our baseline and increase the amount of tumour in steps of 10% to a total of 100% tumour. Looking as Figure 5 this suggests P3, P8 and P9 are the most physiologically relevant parameters as they follow the trend of real data with the highest correlations. Only two appear to change in opposite directions from the measured pulses as the tissue changes from simulated normal to simulated tumour, these are parameters P1 and P6. P1 is the FWHM which may have issues due to the way the simulated pulses deviate from the measured pulses beyond the range from 17.2ps onwards. Similarly, P6, which is related to the shape of the pulse from about 17.2 ps to about 18ps, occurs in the time range in which the measured and simulated pulses deviate most. We note that given that the model is not ideal these plots would need to be repeated with an improved model to increase the confidence in identifying the most relevant parameters for the physiological and compositional changes in the tissue.

## Discussion

There is a close match between the simulation and the measured reflected pulses of liquid water which shows that the FDTD model is highly representative when the spectroscopy data is accurate, and the double Debye theory provides a frequency response very close to the measured frequency response. However, from the comparison of the simulated and measured breast pulses, it can be seen that there are some deviations from the measured pulses that require further investigation, especially the differences in the return to baseline after about 17.5 ps (Figure 3). These deviations suggest that there are some challenges when applying the double Debye theory, which works well with homogenous liquid water, to the more complex composition of biological tissue. Biological tissue exhibits a more inhomogeneous composition and has many physiological differences from sample to sample.

The question of the ability of double Debye theory to represent breast tissue is highlighted by Figure 4, which shows the fit of the double Debye to the real and imaginary components of the permittivity calculated from the breast tissue data. The fit is very good for liquid water, but is not such a good fit for either normal or tumour breast tissue. It is clear that the double Debye model doesn’t exactly match the breast tissue response from the obtained spectroscopy values in these cases.

There are several possible sources for the differences between the simulated and measured breast reflection pulses. The two main assumptions in question are the spectroscopic values (refractive index and absorption coefficients) which may be affected by the referencing and volume correction used to obtain the pure spectroscopy values, and the assumption that double Debye theory represents the dielectric relaxation of breast tissue.

The spectroscopic values of refractive index and absorption coefficient may have errors based on the measurement and calculation methods. Alternative methods of measurement and referencing have been proposed by Huang *et al* [34] which may lead to improved results. Based on the results of liquid water, any inaccuracies in the values due to the measurement of homogeneous samples appear to be small in this case.

Volume correction was used by Ashworth *et al* [29] to obtain pure spectroscopy values for tissue that contains more than one biological component. This correction is required because the tissue samples obtained contain a mixture of tissue types. On average, the proportion of normal tissue in normal samples was 82% (the remaining 18% being adipose tissue), and the proportion of tumour in tumour samples was 92% (the remainder being 5% normal and 3% adipose). Adipose tissue contained on average 90% adipose tissue (the remaining 10% being normal). It should be noted that these are averages and the amounts if different tissues type from sample to sample can vary by a large amount. The frequency dependent refractive index influences the time resolved reflectance and is included in the FDTD model along with the absorption coefficient. As the refractive index and absorption coefficient of adipose tissue is significantly different to that of both tumour and normal tissue; and knowing that normal tissue has the higher adipose content (which is not modelled well with double Debye theory) there is a greater difference between the simulated and actual data for normal tissue.

This volume correction may account for some of the deviation seen between the simulated normal breast pulse and the measured breast pulse, since if the absorption coefficient and refractive index do not represent the pure tissue, on its own, then the double Debye values obtained from the fit to the corrected absorption coefficient and refractive index will not be accurate in representing the pure normal and adipose tissues. The tumour samples are more uniform and homogeneous, at 92% tumour tissue, so the correction could play a smaller part, possibly indicating that the double Debye values are more accurate for this lower lipid content tissue, and this may explain in part the better fit of the modelled tumour pulse to the measured, compared with the normal breast comparison.

Given that the volume correction gives the most accurate method for obtaining the spectroscopic data, and therefore the double Debye values for the individual tissues, we can explore the other possible sources of deviation. It can be seen from Table 1 that some of the Debye values for breast are in the same range as liquid water, especially the static and slower change terms such as ε_S_ and τ_1_, which are within 20%. However for the faster time constant processes, i.e. ε_2,_ ε_∞_ and τ_2_ the values seem to deviate more, up to 60% or more. This indicates that in biological tissue there may still be slower, dominant structural water molecule re-orientation in the electric field, but that the faster single molecule re-orientation is strongly inhibited *in situ* and that there may be other, more complicated processes going on.

The assumption of the double Debye theory applying to breast may be challenged on a number of levels. Firstly, Debye theory applies well to homogeneous media, like water, whereas breast tissue is highly heterogeneous in composition with many physiological and structural elements. Secondly, the Debye theory for water breaks down when the water content is low [35], which is the case for breast tissue which has a high component of adipose lipid (fat) content. Furthermore, the THz region of the spectrum corresponds to a range of frequencies over which several different dielectric relaxation processes may be occurring, so that a more involved theory may be required.

Breast tissue is heterogeneous in its structure, and low in water, especially the lipid portion. Normal breast has around 19% water and tumour slightly more at 26% [36]. This combination of heterogeneity and low water concentration both negate the double Debye approach.

For a more accurate representation of biological tissue, other researchers have used a representation with more terms in the Debye theory, for example triple Debye with three time constants [37], to explain the complicated tissue composition. The Debye theory works reasonably well for frequencies below 1 THz, however, with tissue spectroscopy approaching up to 2 THz, the extra processes going on may require further terms. Liebe *et al* [38] suggested to add two Lorentzian resonant process terms to the double Debye theory to maintain the accurate estimation of their non-linear least square method for frequencies up to 2 THz.

With increased complexity of models comes the need for improvements in the method of fitting the advanced Debye theories to the data as shown by Truong *et al* [37]. They found the existing parameter identification procedures using a nonlinear least square fit far from optimal. To improve the fitting procedure Truong *et al* [39] used the error objective function to solve for the complex nonlinear and non-convex function to fit for the five Debye parameters, as opposed to the nonlinear square based approach. Their approach locates the global optimal solution of the error function minimization, which leads to the optimal double Debye parameters for the interaction between THz and human skin. This method could also be applied to breast data.

However, given the limitations of Debye theory discussed above, a more accurate theoretical representation may be needed. This could be done using a method such as Effective Medium Theory (EMT) and specifically the Bruggeman mixing theory [40], which breaks the problem down into constituent components. As a first approximation, the double Debye theory appears to be useable although limited for representing the THz response of breast tissue. However, even with this limited double Debye model it was possible to simulate the THz pulses for tissue ranging from normal to tumour in 10% increments. From the changes in these parameters we can begin to interpret how changes in tissue composition and pathology may relate to changes in parameters from THz reflection measurements of breast tissue. Aside from simulation parameters relating to the latter time section of the pulse, all the others appear to change in the manner that corresponds to the measured breast pulses. The next stage of this work would be to test other models, and begin to investigate small physiological and compositional changes of tissue using the FDTD. This will begin to elucidate how the changes from normal to tumour influence the THz response of the tissue. Through these studies we may begin to grasp the significance of the heuristic THz parameters we presently use to characterise the tissue and even evolve more specific ones for aiding classifying and differentiating tumour from normal tissue, aid instrument design and measurement practices.

## Conclusion

We have demonstrated that the results from the FDTD model are in good agreement with THz reflection measurements when the double Debye values entered into the model are accurate, as is the case for liquid water. This work corroborates other studies showing the double Debye theory appears well suited for modelling liquid water. The premise that, as biological tissues have a high water content, double Debye theory can also be used to model tissues has been tested. We have shown that at least for breast tissue this approach has limitations due to the complex inhomogeneous nature of biological samples. We have demonstrated the potential of using the double Debye and FDTD methods as a starting point for beginning to understand image contrast parameters and classification of normal and tumour tissue with THz measurements. It may be that other models may improve and deepen this understanding in determining which parameters are physiologically important and how they change with tumour. What this work has allowed us to do is to suggest new avenues for modelling the interaction of THz with breast tissue. One promising direction is to use a dielectric model that is capable of mimicking the spectra of human breast tissue’s complex permittivity. As mentioned previously, breast tissue is heterogeneous and contains adipose (fat) which contains no water and so we would not be expect to model such tissue using the double Debye approach. A non-Debye relaxation model is needed to fit the complex permittivity of adipose tissue, for example Cole-Davidson [41]; which could be combined with the double Debye model to produce a mixture model of human breast tissue; this would allow us to finally begin to understand THz imaging contrast mechanisms in biological samples.
